# Current Mouse Models of Intracranial Aneurysms: Analysis of Pharmacological Agents Used to Induce Aneurysms and Their Impact on Translational Research

**DOI:** 10.1161/JAHA.123.031811

**Published:** 2024-01-23

**Authors:** Dilaware Khan, Xuanchen Li, Tomoki Hashimoto, Rokuya Tanikawa, Mika Niemela, Michael Lawton, Sajjad Muhammad

**Affiliations:** ^1^ Department of Neurosurgery Medical Faculty and University Hospital Düsseldorf, Heinrich‐Heine‐Universität Düsseldorf Düsseldorf Germany; ^2^ Department of Neurosurgery and Neurobiology Barrow Neurological Institute Phoenix AZ USA; ^3^ Department of Neurosurgery, Stroke Center Sapporo Teishinkai Hospital Sapporo Hokkaido Japan; ^4^ Department of Neurosurgery University of Helsinki and Helsinki University Hospital Helsinki Finland; ^5^ Department of Neurological Surgery Barrow Neurological Institute, St. Joseph’s Hospital and Medical Center Phoenix AZ USA

**Keywords:** artery ligation, hypertension, intracranial aneurysm, mouse models, pharmacological treatments, Animal Models of Human Disease, Vascular Biology

## Abstract

Intracranial aneurysms (IAs) are rare vascular lesions that are more frequently found in women. The pathophysiology behind the formation and growth of IAs is complex. Hence, to date, no single pharmacological option exists to treat them. Animal models, especially mouse models, represent a valuable tool to explore such complex scientific questions. Genetic modification in a mouse model of IAs, including deletion or overexpression of a particular gene, provides an excellent means for examining basic mechanisms behind disease pathophysiology and developing novel pharmacological approaches. All existing animal models need some pharmacological treatments, surgical interventions, or both to develop IAs, which is different from the spontaneous and natural development of aneurysms under the influence of the classical risk factors. The benefit of such animal models is the development of IAs in a limited time. However, clinical translation of the results is often challenging because of the artificial course of IA development and growth. Here, we summarize the continuous improvement in mouse models of IAs. Moreover, we discuss the pros and cons of existing mouse models of IAs and highlight the main translational roadblocks and how to improve them to increase the success of translational IA research.

**Nonstandard Abbreviations and Acronyms**
IA: intracranial aneurysm

Aneurysmal subarachnoid hemorrhage (aSAH), with a crude worldwide incidence of ≈7.9 per 100 000 person‐years,^1^ is a potentially devastating cerebrovascular disease. Despite appropriate surgical and medical care, the mortality rate of aSAH is high (40%).^2^, ^3^ In about 15% of SAH cases, the patient suffers sudden death and cannot receive any medical intervention. Most of the survivors of aSAH suffer from physical and mental disabilities and hence pose a high socioeconomic burden at the community level.^4^

The intracranial aneurysm (IA) is defined as an outward bulging of the vessel wall with a diameter of >150% of the parent vessel.^5^, ^6^ The prevalence of unruptured IAs is 3% to 5% in the general population, and the incidences are slightly higher in women.^7^, ^8^ Epidemiological data demonstrate that arterial hypertension, female sex, age, chronic alcohol consumption, smoking, and family history of aSAH in first‐degree relatives are the main risk factors behind IA development and progression.

Both endovascular coiling and surgical clipping of IAs have treatment risks of 4.8%^9^ and 6.7%^10^ unfavorable outcomes, respectively. This urges us to explore novel mechanistic‐based pharmacological targets to block the formation and progression of IAs. The pharmacological agents such as statins, cerebrolysin, dapsone, corticosteroids, calcium channel blockers, magnesium, antifibrinolytics, aspirin, epoxide hydrolase, and inhibitors of cyclooxygenase and thromboxane A2 synthase have been used in clinical studies. But the outcome was not favorable to a tangible degree.^11^, ^12^ Unfortunately, to date, there is no single valid pharmacological option to treat these lesions.

There is an urgent need to explore and validate basic key mechanisms underlying IA formation, progression, and rupture pathophysiology to find suitable pharmacological targets. Multiple animal models have been recently developed to study molecular and cellular pathophysiological mechanisms and to search for and develop novel pharmacological treatments. Using suitable animal models to efficiently translate preclinical data into the clinical setting is critical. This systematic review summarizes existing knowledge on IA mouse models and discusses translational research issues.

## Methods

### Systematic Literature Search

The literature search was performed according to Preferred Reporting Items for Systematic Reviews and Met‐Analyses (PRISMA) guidelines. The search for articles describing/using IA mouse models was conducted in March 2023 in PubMed, Bielefeld Academic Search Engine (BASE), and Embase. The detailed search strategy was *Murine or mouse or mice model of intracranial aneurysm* and *Cerebral aneurysms murine or mouse or mice* and *Ruptured cerebral aneurysm murine or mouse or mice* and *Intracranial aneurysms murine or mouse or mice* and *aSAH murine or mouse or mice* and *Unruptured cerebral aneurysms murine or mouse or mice* and *Unruptured cerebral aneurysms murine or mouse or mice*. All results were deduplicated, and irrelevant research articles were screened after reading titles.

**Figure 1:**
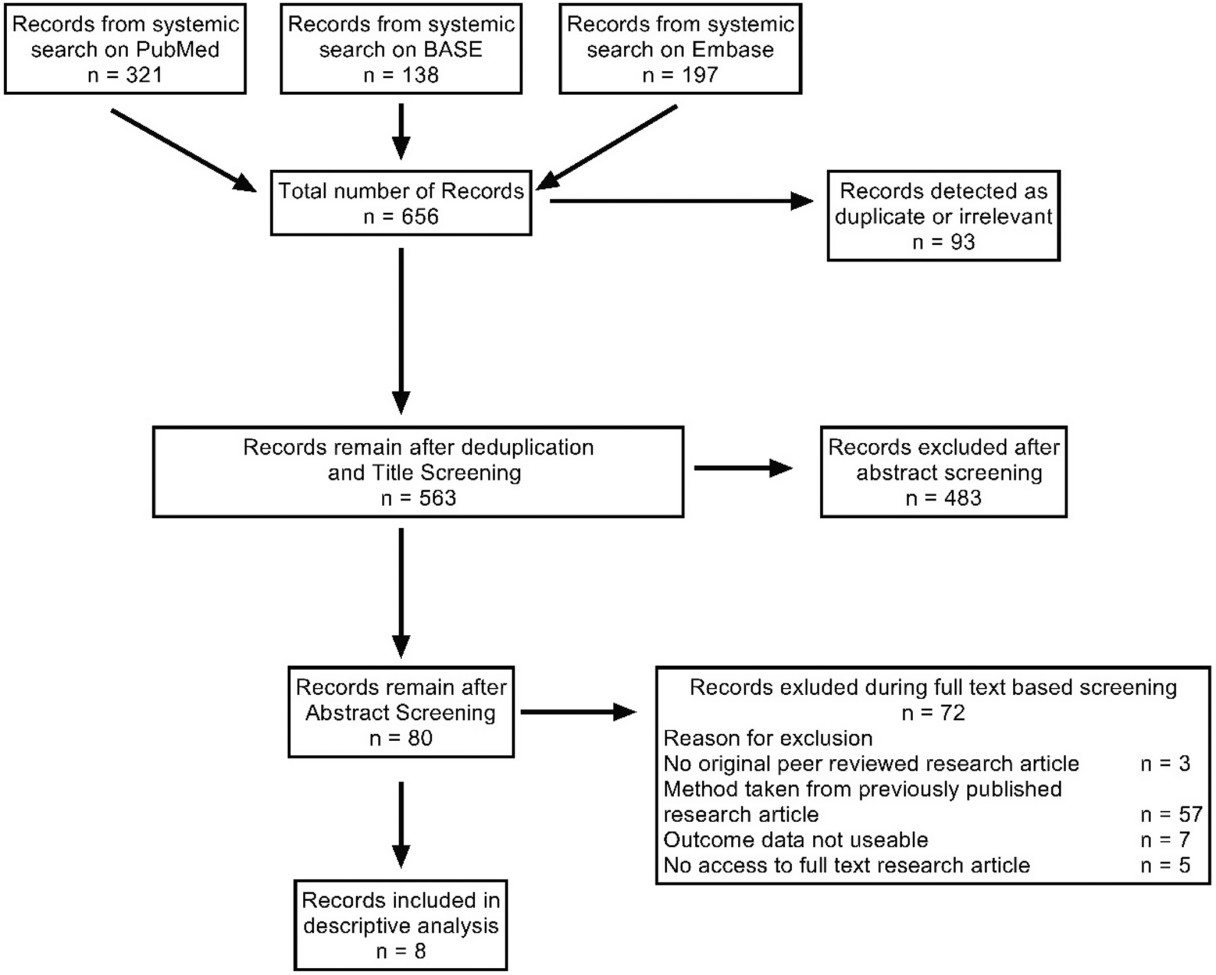
Flowchart showing a systematic search of the literature. A systematic literature search was performed in March 2023. BASE indicates Bielefeld Academic Search Engine.

The systematic search on PubMed, BASE, and Embase for literature using an IA mouse model delivered 656 records. Five hundred sixty‐three unique research articles remained after deduplication and title screening. After the abstract screening, 80 research articles were left for full‐text screening. Eight research articles were included in the descriptive analysis after full‐text screening. The most frequent reason for exclusion was adopting methods from previously published research articles. The systematic search literature flow chart is shown in Figure 1.

### Literature Screening Criteria

The original peer‐reviewed research articles describing the first time the method for the induction of IAs and reporting the usable data (number or percentage of mice with IA formation or rupture) using wild‐type mice were included. The reviews, book chapters, thesis, and research articles in foreign languages were excluded from the abstract screening. The research articles that adapted the IA mouse model from the previously published research articles or made changes only in the concentration of pharmacological compounds were not considered the first research articles describing the IA mouse model; thus, these research articles were excluded from the descriptive analysis.

### Animal Models of IAs

IAs rarely occur naturally in laboratory animals. Therefore, using diverse strategies, various animal models of IAs^13^ have been created in different species, including mice, rats, rabbits, swine, dogs, sheep, and primates.^14^, ^15^ Each IA animal model comes with its advantages and limitations. Consequently, different animal models are used for different purposes. Usually, large animals like canines and pigs are used to investigate different therapeutic approaches and training interventionists.^14^ In addition to that, extracranial aneurysms generated in large animals are used to test endovascular treatment options. The models of extracranial aneurysms have been reviewed in detail elsewhere.^16^ Small animals like rabbits and rodents are used mainly to study mechanisms of IA development, progression, and rupture.^17^

### Current Mouse Models of IAs

The first rodent model of IAs was developed in rats by Hashimoto et al,^18^ which was later adapted into a mouse model of IAs.^19^ The hypothesis was that IAs are formed because of hemodynamic stress at fragile arteries. The original model by Hashimoto et al used common carotid artery ligation to increase hemodynamic stress and ligation of posterior branches of renal arteries with enhanced salt intake to induce renal hypertension. In addition, rats were treated with β‐aminopropionitrile. β‐aminopropionitrile alone can cause significant changes to arteries and can induce aortic aneurysm rupture.^20^ β‐aminopropionitrile is a lysyl oxidase inhibitor, which weakens the arterial walls by inhibiting the cross‐linkage of collagen and elastin fibers.^21^, ^22^ At the bifurcation sites of cerebral arteries, IAs developed spontaneously, which showed pathologic features (deterioration of internal elastic lamina and loss of smooth muscle cell layer) like human IAs.^23^ Because many modifications have been introduced into the rodent IA model, the most frequent alterations were made in the method of hypertension induction in rodent models. The induction of hypertension was achieved either alone or with a combination of renal artery ligation, deoxycorticosterone administration, and high‐salt diet intake.^13^ Most studies use β‐aminopropionitrile treatment and ligation of the common carotid artery and renal artery to induce IAs in rats.^13^

**Figure 2:**
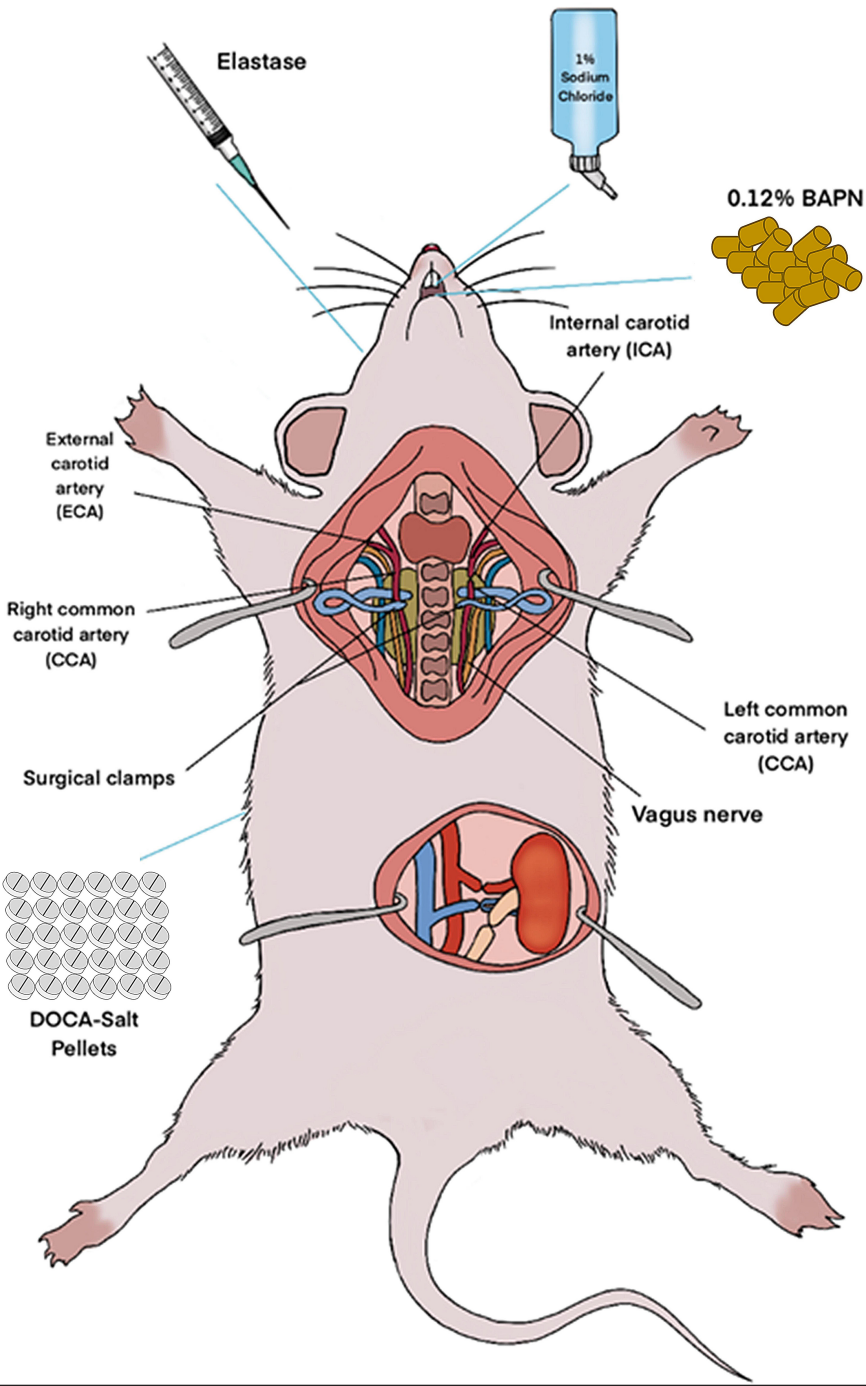
Induction of intracranial aneurysms in mice using a combination of different surgical and pharmacological interventions. BAPN indicates β‐aminopropionitrile; and DOCA, deoxycorticosterone.

Mouse models of IAs have been generated by applying different approaches (Figure 2; Table). In 2002, Morimoto et al successfully induced IAs in mice by ligation of the left common carotid artery and induced hypertension by ligation of posterior branches of bilateral renal arteries with increased salt (1% NaCl) intake.^19^ This model is the adaptation of the rat model of IAs developed by Hashimoto et al^18^ in mice. It is not fundamentally different in terms of the manipulations. This mouse model used the same hypertension model as used in the IA rat model of Hashimoto et al. Similar to IAs in humans, these aneurysms showed internal elastic lamina fragmentation, smooth muscle cell layer thinning, and adventitial tissue degeneration.^19^ Sadamasa et al modified the Morimoto et al IA mouse model by increasing the salt concentration (8% NaCl) intake and adding 0.12% β‐aminopropionitrile to the food.^24^ Both studies evaluated IA formation 4 months after the operations and classified aneurysmal changes into early stage and advanced stage.^19^, ^24^ The advanced stage aneurysms increased from 43% in the Morimoto et al mouse model^19^ to 50% in the Sadamasa et al mouse model.^24^ Because of the differences in the circle of Willis between rats and mice, the size and location of IAs are different. The mouse circle of Willis is incomplete; the anterior circulation is separated from the posterior circulation. That is why we do not see as many aneurysms in the posterior circulation in the IA mouse model as observed in the posterior circulation in the IA rat model. One limitation of these IA mouse models is that it takes 4 months to develop IAs, and the second limitation is that the mouse models by Morimoto et al and Sadamasa et al produce microscopic IAs or aneurysmal changes.^19^, ^24^ In contrast, the rat model induces large IAs.

**Table 1:** Models of IAs in Mice

Study	Method	Mice used/mice died	Sex	Duration of IA induction/rupture	Features	Reference
1	Ligation of the left common carotid artery and posterior branches of right and left renal arteries, 1% saline	20 Mice used/2 mice died	Male	4 mo	Fragmentation of internal elastic lamina, degeneration of adventitial tissue, and smooth muscle cell layer thinning. Incidence of aneurysm formation in 77% of animals. Observations were made on ACA‐OA bifurcations.	Morimoto et al, 2002, *Stroke* ^19^
2	Ligation of left common carotid artery, posterior branches of right and left renal arteries, 8% NaCl, 0.12% β‐aminopropionitrile	8 Mice used	Male	4 mo	Incidence of aneurysm formation in 75% of animals. Observations were made on ACA‐OA bifurcations.	Sadamasa et al, 2003, *Stroke* ^24^
3	Elastase (0–35 mU), angiotensin II‐induced hypertension	44 Mice used		2 wk	Discontinued endothelial cell layer, severely disorganized elastic lamina, macrophage infiltration into the aneurysmal wall, IAs 3–5 times larger than parent artery. Seventy‐seven percent of mice developed IAs along the CoW or its major branches.	Nuki et al, 2009, *Hypertension* ^6^
4	DOCA‐salt–induced hypertension, elastase (25–35 mU)	8 Mice used for IA induction, 8 mice used for IA rupture	Male	IA induction for 7 d, IA rupture for 28 d	IA formation in >60% and IA rupture in 50%–60% of mice.	Makino et al, 2012, *Stroke* ^25^
5	Ligations of the left common carotid artery and right renal artery, angiotensin II (1000 ng/kg per min) SC, elastase (5, 10, or 20 μL of 10 U/mL or 10 μL of 1 U/mL), 8% sodium chloride, and 0.12% β‐aminopropionitrile	40 Mice used, 10 mice for each dose of elastase	Female	3 wk	Infiltration of inflammatory cells, loss of endothelial cells, thickening of smooth muscle cell layer in aneurysmal wall. Mice (90%–100%) developed aneurysms and incidence of ruptured aneurysm in (20%–60%) mice depending on elastase concentration and amount. Observations were made within the CoW	Hosaka et al, 2014, *J Neurointerv Surg* ^26^
6	DOCA‐salt–induced hypertension, 0.12% β‐aminopropionitrile, elastase (35 mU)	22 Mice used	Male	28 d	Histological changes similar to humans, a layer of discontinuous endothelial cells, disorganized elastic lamina. IA formation in 81.8% of mice. Ruptured IAs in 68.2% of mice.	Starke et al, 2014, *J Neuroinflammation* ^5^
7	Ligation of left common carotid artery, elastase (10 μL of 1 U/mL elastase in phosphate buffered saline), angiotensin II (1000 ng /kg per min) SC, 8% NaCl, 0.12% β‐aminopropionitrile	12 Mice used	Female	21 d	IAs formation in 66.7% of mice. Ruptured IAs in 62.5% of mice. Observations were made within the CoW.	Patel et al, 2021, *Cureus* ^41^
8	DOCA‐salt–induced hypertension, elastase, NaCl (10 g/L), and KCl (2 g/L)	25 Mice used	Male	21 d	Macrophage infiltration into the aneurysmal wall. IA formation in 96% of mice. Ruptured IAs in 88% of mice.	Jiang et al, 2021, *J Drug Target* ^37^

ACA‐OA indicates anterior cerebral artery‐olfactory artery; CoW, circle of Willis; DOCA, deoxycorticosterone; and IA, intracranial aneurysm.

Hashimoto's group combined 2 factors associated with human IAs, hypertension and the elastic lamina disruption, to induce IA formation. Nuki et al developed an IA mouse model by injecting 35 mU elastase into the right basal cistern to weaken the arterial walls and angiotensin II (1000 ng/kg per minute) was injected subcutaneously to induce hypertension, which led to formation of IAs in 77% of the mice within 2 weeks.^6^ The IAs were ≈3 to 5 times larger than their parent arteries, and the formed IAs closely resembled human IAs.^6^ A discontinued endothelial cell layer with scattered faint smooth muscle cell staining was observed in the thick vascular wall of IAs.^6^ Severely disorganized internal elastic lamina was observed in thin and thick portions of the artery.^6^ The dose of elastase and angiotensin II affected the number of IAs formed.^6^ The model of Nuki et al suffered from high preoperative mortality because of the rapid rise in blood pressure in the angiotensin II‐induced hypertension model and severe vascular inflammation caused by angiotensin II. That is why, in the next IA mouse model, to reduce preoperative mortality, Makino et al used the deoxycorticosterone–salt hypertension model for induction of IAs in mice.^25^

Makino et al developed the IA mouse model with ruptured aneurysm using a single elastase (25–35 mU) injection into the right basal cistern and deoxycorticosterone–salt to induce hypertension. One week after the nephrectomy, a deoxycorticosterone pellet was implanted subcutaneously, and animals were fed 1% NaCl in drinking water. In this mouse model, 60% of the mice developed aneurysms. The formation of IAs occurred during the first week following IA induction. The rupture of IAs occurred in 60% of the mice during the second and third week after IA induction.^25^ An aneurysmal rupture results in subarachnoid hemorrhage that causes neurological symptoms (ie, motor deficits or death), which can be detected by performing a simple daily neurological examination.

Multiple groups have used this model in various strains of mice and other species with or without modifications.^5^, ^26^, ^27^, ^28^, ^29^, ^30^, ^31^, ^32^, ^33^, ^34^, ^35^, ^36^ This model has been extensively used to study mechanisms for the development of IA rupture. In addition, many independent groups have used this model to test various pharmacological agents to prevent IA rupture.^25^, ^34^, ^37^, ^38^, ^39^, ^40^

Later, Hosaka et al modified this model by using left common carotid and right renal artery ligation; 1 week later, elastase (5, 10, or 20 μL of 10 U/mL or 10 μL of 1 U/mL) was injected into the right basal cistern. At the same time a micro‐osmotic pump containing angiotensin II (1000 ng/kg per minute) was implanted into a subcutaneous pocket, and mice were fed hypertensive food containing 8% NaCl and 0.12% β‐aminopropionitrile.^26^ Depending on the concentration and amount of elastase, 90% to 100% of mice developed IAs, and 20% to 60% of mice had ruptured IAs. Infiltration of inflammatory cells (CD [cluster of differentiation]‐45 positive), especially macrophages (F4/80‐positive cells) in aneurysmal walls with loss of intimal endothelial cells and thickening of a smooth muscle cell layer in aneurysmal walls was revealed, which is similar to human IAs.^26^ Recently, Patel et al further modified the model generated by Hosaka et al, and showed that right renal artery ligation did not affect the incidence of IA formation and rupture.^41^ Starke et al developed the IA mouse model by using nephrectomy, and 1 week later, a deoxycorticosterone pellet was implanted, 1% NaCl in water was started with 0.12% β‐aminopropionitrile, and elastase (35 mU) was injected into the right basal cistern.^5^ IAs were formed in 81.8% of the mice, and the incidence of ruptured IAs was observed in 68.2% of mice.^5^ Similar to histological changes in IAs in humans, these IAs presented layers of discontinuous endothelial cells and disorganized internal elastic lamina. The IA mouse model developed by Jiang et al was similar to the IA mouse model developed by Makino et al with 1 exception, which was that Jiang et al included KCl (2 g/L) in salt intake.^25^, ^37^ This modification in salt intake increased the percentage of mice developing aneurysm formation from 60% to 96% and the percentage of mice presenting ruptured aneurysm from 50% to 60% to 88%.^25^, ^37^ An increase in number of infiltrated F4/80‐positive macrophages was observed in IA tissue in a mouse model described by Jiang and colleagues.^37^ Most studies use the cisternal elastase injection model to induce IAs in mice.^13^

## Discussion

Currently, existing IA mouse models need a combination of surgical and pharmacological interventions to generate hypertension‐induced wall shear stress and weaken cerebral vessels with the ultimate goal to achieve induction of IAs with high incidences (Figure 2; Table). The preference for an animal model depends on various factors, including ethical considerations, resources, study duration, and reproducibility. However, the research question of the study is the most important factor to be considered. Each animal model has its limitations. For ease, research questions can be divided into 2 types. The first type of research question deals with investigating the mechanisms leading to the induction, development, progression, and rupture of IAs. The second type of research question deals with developing specific therapeutic tools and their use against the formation and rupture of IAs. State‐of‐the‐art laboratories worldwide use large animals, such as swine, sheep, and canines, to address the second type of research questions. However, small animals, such as rabbits and rodents (rats and mice), are feasible tools to investigate mechanisms of disease pathology.

The use of rodents has many advantages. Rodents use small spaces and need fewer resources to keep them species appropriate. Unlike large animals, they do not need specific operation theaters, handling, anesthetic setup, postoperative housing, and monitoring. Moreover, the training of new researchers is much faster and easier.

The rat model of IAs is reliable to study the time course for the development of IAs. Since its first development, multiple modifications have been introduced to the targeted parameters. The mouse model was developed much later, but it has some advantages over the rat model. Due to the availability of genetic modifications at a larger scale in different mice strains, mice can be used to study the specific role of a particular gene.

For a better understanding of the prognosis of IA formation, 2 basic factors are needed to be considered: the biomechanical stimuli on the arterial walls asserted by blood flow and changes that would cause the arterial wall remodeling. Both of these factors work synergistically, leading to IA formation and eventually rupturing.^42^ In human and animal studies, hemodynamic stress has been observed to contribute to IA formation.^17^, ^43^, ^44^, ^45^ In animal experiments, weakening of arterial walls has been shown to affect IA formation and rupture.^6^, ^18^, ^26^ Both increased hemodynamic stress and the weakening of arterial wall can be achieved by applying different strategies. All of the pharmacological agents used to induce hemodynamic stress or to weaken arterial walls have additional ramifications, which can interfere with the research question that needs to be addressed in the study. Therefore, it is important either to use appropriate pharmacological agents to increase hemodynamic stress and weaken the arterial wall for specific questions or to consider the additional effects of these pharmacological agents.

Hemodynamic stress is induced by using a combination of surgical strategies, including common carotid artery ligation, bilateral renal artery ligation, unilateral nephrectomy, and administration of pharmacological agents, including deoxycorticosterone or angiotensin II with increased NaCl in drinking water or food.^5^, ^6^, ^19^, ^25^, ^26^, ^41^, ^46^

The renin‐angiotensin‐aldosterone system is a hormone system that regulates blood pressure and water balance. Renin released by arterial cells in the kidney is converted to angiotensin I in the liver, which is carried to the lungs, where it is converted to angiotensin II. Angiotensin II is a potent vasoactive peptide, which increases blood pressure by constricting blood vessels. It also results in increased aldosterone release that, in turn, increases the reabsorption of sodium and water into the blood, thus causing hypervolemia, which increases blood pressure, leading to hypertension. In the mouse models of IAs, either angiotensin II or deoxycorticosterone (a precursor of angiotensin II) is administered.^5^, ^6^, ^25^, ^26^, ^41^, ^46^

Angiotensin II exerts proinflammatory effects on leukocytes, endothelial cells, and smooth muscle cells.^47^, ^48^, ^49^ Angiotensin II activates NF‐κB (nuclear factor kappa light chain enhancer of activated B cells)^50^, ^51^ and upregulates the expression of proinflammatory cytokines IL (nterleukin)‐1β, IL‐6, TNF (tumor necrosis factor)‐α,^49^, ^52^ proinflammatory receptors (TLR [toll like receptor]4),^49^, ^53^ chemokines (MCP [monocyte chemoattractant protein]‐1 and IL‐8),^47^, ^48^ adhesion molecules (ICAM [intercellular adhesion molecule]‐1, VCAM [vascular cell adhesion molecule]‐1, P‐selectin, E‐selectin),^54^ and MMPs (matrix metalloproteinases; MMP1, MMP2, MMP9).^48^, ^49^ All of these factors increase cellular and molecular inflammation and are known to induce the formation, progression, and rupture of the IAs.^5^, ^36^, ^55^, ^56^, ^57^, ^58^, ^59^ Furthermore, angiotensin II stimulates the release of aldosterone via acting on the adrenal cortex. It should be noted that aldosterone itself mediates inflammation and induces oxidative stress in vessels.^60^, ^61^ Therefore, regulation of these molecules by angiotensin II should be considered when investigating the same underlying molecular mechanisms in IA pathophysiology. High salt intake has also been associated with inflammation.^62^ High salt intake has been reported to promote T helper 17 cell differentiation and activation^62^, ^63^, ^64^ and decrease regulatory T cells.^63^ It also increases the production of TNF‐α and IL‐2 in T helper 17 cells.^62^ High salt in in vivo mice experiments and in vitro cell culture models increased the expression of proinflammatory mediators (IL‐1‐β, IL‐6, IL‐8, TNF‐α, MCP‐1, MCP‐2, CXCL [chemokine (C‐X‐C motif) ligand]1, CXCL2, IL‐17, CCR2 [C‐C motif chemokine receptor 2], TLR3, TLR4 and CD14)^63^, ^65^, ^66^ and decreased the expression of anti‐inflammatory mediators (IL‐10, CCL [C‐C motif chemokine ligand ]18, CCL22).^63^, ^66^ Moreover, it increased the expression of VEGF (vascular endothelial growth factor).^65^ Therefore, in addition to shear stress, the proinflammatory and oxidative effect of angiotensin II and increased salt intake should be considered while investigating anti‐inflammatory interventions in a mouse model of the IAs. Moreover, angiotensin II and high salt promote endothelial dysfunction,^67^, ^68^ which consequently increases the expression of proinflammatory molecules and MMPs^69^, ^70^, ^71^, ^72^ that are known to contribute to IA pathophysiology^5^, ^55^, ^58^; therefore, while using an animal model using angiotensin II or deoxycorticosterone to study endothelial dysfunction in IAs formation, the direct effect of angiotensin II and high salt on endothelial cells should be considered.

Elastase and β‐aminopropionitrile are used to weaken the cerebral arteries. Elastase is directly injected into the basal cistern,^5^, ^6^, ^25^, ^26^, ^37^, ^41^ whereas β‐aminopropionitrile is added to the food.^5^, ^24^, ^26^, ^41^ β‐aminopropionitrile weakens the arterial wall by inhibiting the cross‐linkage of collagen and elastin.^21^, ^22^ The incidence of IAs formation is elastase dose–dependent.^6^, ^26^ Moreover, the use of elastase significantly decreases the duration for IA induction and is used in all models of IA rupture.^5^, ^6^, ^25^, ^26^, ^37^, ^41^ Elastase is a serine protease, which is also endogenously produced by different cell types, including neutrophils, macrophages, endothelial cells, and smooth muscle cells.^73^, ^74^, ^75^, ^76^ The neutrophil elastase induces endothelial cell apoptosis,^77^ promotes inflammatory synthetic phenotype in smooth muscle cells,^78^ and supports macrophage cell adhesion.^79^ Some studies show the proinflammatory effect of elastase, where it increases the expression of inflammatory cytokines (IL‐1β, IL‐6, IL‐8, TNF‐α)^79^, ^80^ and MMP2^80^ in macrophages, whereas other studies show its anti‐inflammatory effect, where it degrades inflammatory cytokines TNF‐α, IL‐1β, IL‐6, and IL‐8.^81^, ^82^, ^83^ Moreover, elastase inhibited NF‐κB translocation to the nucleus and cleaved TLRs on macrophages.^83^ In smooth muscle cells, elastase suppresses IL‐8 expression and release.^84^ It can be suggested that, on the one hand, the inflammatory effect of elastase increases the expression of these inflammatory mediators and MMPs. On the other hand, the protease function of elastase causes the degradation of these inflammatory mediators. Furthermore, using different experimental setups and models can lead to varying results for target parameters. Nevertheless, these findings suggest that elastase can modulate inflammation in various ways, which is of high importance when investigating inflammation in the experimental models of IAs using elastase to induce IAs; these mechanisms should be considered seriously. When elastase is used in combination with β‐aminopropionitrile, it increases the rate of IA formation and rupture.^5^, ^25^ We could not find reports suggesting that β‐aminopropionitrile modulates inflammation. The effect of elastase is local, but it modulates inflammatory response and requires a stereotactic injection into the basal cistern, whereas β‐aminopropionitrile most probably does not interfere with main inflammatory pathways and can be administered through food, but its effect can be generalized.

The sex of the animals is another relevant factor. Previously published research articles have established a causal relationship between estrogen and the formation and rupture of IAs.^38^, ^85^ Tada et al showed that ovariectomized mice had a higher incidence of IA formation.^85^ Estrogen treatment reduced the incidences of IAs formation and rupture.^38^, ^85^ Furthermore, they also reported that stimulation of estrogen receptor‐β provided protection against IA formation.^38^, ^85^ The female hormone estrogen has an anti‐inflammatory effect,^86^ and it inhibits endothelial dysfunction.^87^ It also accelerates the resolution of inflammation and promotes M1‐M2 transition in the macrophage cell culture model,^86^ which can consequently provide protection against IA rupture.^59^ Through these mechanisms, estrogen exerts a protective effect against aneurysm formation and rupture in women.^38^, ^40^ On the contrary, the male hormone testosterone has been shown to exacerbate cerebral vascular injury in mouse model of IAs.^88^

Furthermore, there should be some distinctions between the IA model that can be used to study only IA formation and the IA model that can be used to investigate the mechanism of rupture. The IA mouse model developed by Sadamasa et al^24^ seems to be a better choice to dissect the mechanism underlying IA formation. The IA rupture model is clinically relevant and has more value in translational aspects. The IA mouse model modified by Patel et al^41^ is the model of choice to investigate molecular and cellular mechanisms leading to IA rupture. This is a simple model, and from an animal welfare point of view, it exerts less burden on animals while keeping the key elements of the IA model intact.^41^ However, it uses angiotensin II, which causes high preoperative mortality by inducing rapid blood pressure rise and severe vascular inflammation. Therefore, future research should focus on finding alternative methods to induce blood pressure with similar effect but less mortality. The IA mouse models developed by Jiang et al^37^ and Starke et al^5^ are currently better available alternatives to study IA rupture in mice. We would suggest considering the IA mouse model modified by Jiang et al^37^ as the first option, because in this model the number of IAs formed and rupture is higher than in the IA mouse model developed by Starke et al.^5^ Also, the IA mouse model by Jing et al^37^ takes less duration for the formation and rupture of IAs than the IA mouse model by Starke et al.^5^ Taken together, multiple factors, particularly the use of pharmacological agents to induce IAs, should be considered for a particular research question, with the overarching goal to improve translational research in the field of IAs.

## Conclusions

The pharmacological agents used to induce IAs in mice have additional ramifications, which can interfere with the underlying mechanism being investigated. Therefore, the pharmacological agents and their side effects should be considered to improve clinical translation.

## Sources of Funding

This work was supported by grants from Peek and Cloppenburg Stiftung 2021, Bundesministerium für Bildung und Forschung and Forschungskommission Heinrich‐Heine University Düsseldorf 2020 and 2022 to S. Muhammad; and R01NS109584, R01AG07780, and R01NS109382 from the National Institutes of Health, Barrow Neurological Foundation, and Brain Aneurysm Foundation to T. Hashimoto.

## Disclosures

None.
